# A mouse model of oxycodone reward following its oral self-administration in a sweetened solution

**DOI:** 10.3389/fnbeh.2026.1862996

**Published:** 2026-07-27

**Authors:** Shayesteh Rounama, Prince Meziem, Abdul Hamid, Iffat H. Era, Kabirullah Lutfy

**Affiliations:** 1 California State Polytechnic University, Pomona, CA, United States; 2 Department of Biotechnology and Pharmaceutical Sciences, College of Pharmacy, Western University of Health Sciences, Pomona, CA, United States

**Keywords:** oxycodone, conditioned place preference (CPP), reward, reinforcement, oral self-administration, mu opioid receptor (MOP), male and female, knockout mouse

## Abstract

**Introduction:**

This study aimed to develop a preclinical model in which reinforcement and reward can be measured in the same subject. To this end, we combined the power of drug self-administration and place conditioning paradigms and determined whether voluntary oral oxycodone self-administration would induce conditioned place preference (CPP) and whether this response could be extinguished and reinstated. To assess the face validity of the model, we also evaluated the role of the mu-opioid receptor (MOP) in this process.

**Methods:**

Male and female C57BL/6 mice, as well as male MOP knockout mice and their wild-type littermates, were initially conditioned with sucrose (4%) in both conditioning chambers for 1 h daily for 2 weeks (4 days a week). Mice were then tested for basal place preference and then underwent alternate-day conditioning (1 h daily) with sucrose (4%) in one chamber and oxycodone in a 4% sucrose solution in the opposite chamber the following day for four consecutive days, followed by a CPP test. The following week, another set of conditioning and testing for CPP was repeated. Subsequently, mice were exposed to the sucrose (45) solution alone for 4 days each week, and a test for extinction of the CPP response at the end of each week. After extinction, mice were challenged with oxycodone (5 mg/kg) and tested for the reinstatement of CPP.

**Results:**

Both male and female mice demonstrated robust voluntary oral oxycodone self-administration in a 4% sucrose solution, but it was lower in females than in males. Oxycodone self-administration was associated with CPP in male and female mice. This response was extinguished and reinstated. Unlike wild-type mice, the CPP response was not observed in mice lacking MOP, suggesting that MOP is involved in this response.

**Discussion:**

This is the first study providing evidence for the development of a translational mouse model of reward utilizing oral oxycodone self-administration in a sweetened solution.

## 1 Introduction

Prescription opioids continue to be a major public health issue across the US, as evidenced by a total of over 108,000 reported overdose deaths in 2022, with 76 percent of those deaths attributed to opioid overdoses (CDC, 2023). Oxycodone, one of the several prescription opioids, is also highly linked to both opioid use disorders and overdose deaths. Additionally, there is a high societal cost from the misuse of prescription opioids, with estimated annual expenditures for health care, law enforcement, and loss of productivity reaching $78.5 billion in 2013 (Florence et al., 2016). Individuals misusing prescription opioids are also significantly more likely to begin using heroin than non-misusers of prescription opioids, indicating a strong link between the two opioids (Cicero et al., 2013; Jones, 2013).

Oxycodone is a semi-synthetic opioid analgesic and acts primarily as a mu opioid receptor (MOP) agonist (Olkkola et al., 2013). Indeed, oxycodone possesses rewarding and reinforcing actions in rodents (Babb et al., 2023; Brice-Tutt et al., 2023; Collins et al., 2016; Fan et al., 2019; Niikura et al., 2013; Socha et al., 2024). Furthermore, C57BL/6J mice (Walentiny et al., 2019) as well as non-human primates (Withey et al., 2020) discriminate between oxycodone and vehicle. In recreational opioid users, intravenous oxycodone has been associated with liking and high (Backonja et al., 2016), suggesting that, like other opioid agonists, it has a high potential for abuse. While oxycodone is a major player in today’s opioid epidemic, research examining the rewarding effects of oxycodone in animal models is limited compared to that of other opioids, such as morphine. Research into the rewarding effects of opioids in animal models often utilizes experimenter-administered drug delivery methods, i.e., subcutaneous or intraperitoneal administration. However, epidemiologic studies indicate that prescription opioid misuse occurs most frequently through oral ingestion of oxycodone, and that some individuals will continue to ingest prescription opioids orally even after they have access to abuse-deterrent formulations (Cicero et al., 2014). Therefore, it is important to develop preclinical models of opioid reward that utilize voluntary oral self-administration of the test drug to increase the translational relevance of the results.

The place conditioning paradigm, a Pavlovian conditioning paradigm, is widely used to evaluate the motivational and rewarding effects of abused drugs. In this paradigm, subjects learn to associate the interoceptive effects of a drug with a unique environmental context and thus express a preference for the drug-paired environment, i.e., they exhibit a conditioned place preference (CPP) response when tested under a drug-free state (Bardo and Bevins, 2000; Bardo et al., 1995; Tzschentke, 2007). The place conditioning paradigm is a valuable approach to study the acquisition of CPP, its extinction, and reinstatement after re-exposure to the drug, including oxycodone (Babb et al., 2023; Brice-Tutt et al., 2023; Collins et al., 2016; Niikura et al., 2013; Socha et al., 2024). The use of CPP provides an opportunity to evaluate the rewarding effects of opioids and the likelihood of relapses in response to re-exposure to the drug, because CPP involves associative learning and memory processes. Therefore, this study was designed to develop a preclinical model of reward employing voluntary oral oxycodone self-administration in mice to assess whether oral oxycodone self-administration would be associated with reward-related behavior. We examined the acquisition, extinction, and reinstatement of oxycodone-induced CPP in male and female C57BL/6J mice as well as in male mice lacking MOP and their wild-type littermates.

## 2 Materials and methods

### 2.1 Subjects

Male and female C57BL/6J mice (12–16 weeks of age at the start of experiments) were used for the first experiment. Male mice lacking the mu opioid receptor (MOP), generated by deletion of exon 2 of the *Oprm1* gene (Matthes et al., 1996), and their wild-type littermates were used to assess the role of MOP in the rewarding action of oxycodone. One week before the start of conditioning, mice were housed individually in a temperature- (22 ± 1 °C) and humidity-controlled vivarium under a 12 h light/12 h dark cycle (lights on at 07:00 am), with *ad libitum* access to standard laboratory chow and water except during behavioral testing sessions. Animals were housed individually to acclimate to the novel environment, as they are housed in groups. This was also necessary to measure their intake accurately and, importantly, prevent fighting and social defeat in mice if they are housed together after each conditioning day. All procedures were approved by the Institutional Animal Care and Use Committee (IACUC; protocol R24/IACUC/031) and were conducted in accordance with the NIH guidelines for the proper care and use of animals in research.

### 2.2 Drugs

Oxycodone hydrochloride was purchased from Sigma-Aldrich (St. Louis, MO, USA). For oral self-administration, oxycodone was dissolved in a 4% (w/v) sucrose solution prepared in mouse drinking water. Sucrose ( ≥ 99.5%) was also obtained from Sigma-Aldrich. Control (vehicle) solutions consisted of 4% sucrose without oxycodone. The working dose of oxycodone was 16 mg/kg. This dose was selected based on our pilot dose–response study showing that this dose of oxycodone induces a robust CPP response. To ensure accurate dosing via voluntary consumption, oxycodone concentration was individualized for each mouse based on their body weight and the volume of sucrose they consumed on the last day of the second week of the habituation period. This procedure ensured that mice voluntarily consumed an oral dose of 16 mg/kg oxycodone during conditioning sessions.

### 2.3 Experimental design

#### 2.3.1 To establish a mouse model of voluntary oral oxycodone self-administration and conditioned place preference in the same mice

The place conditioning paradigm was used to determine whether voluntary oral oxycodone intake produces conditioned place preference (CPP), a model of reward (Bardo and Bevins, 2000; Bardo et al., 1995; Tzschentke, 2007). The conditioning and testing for place preference, its extinction, and reinstatement were conducted in a three-chambered place conditioning apparatus (MedAssociates, Fairfax, VA, USA), as described by our group previously (Nguyen A. T. et al., 2012). We used an unbiased and counterbalanced place conditioning paradigm, in which there was no initial preference toward one conditioning chamber over the other. Also, some mice received oxycodone in 4% sucrose solution in the black chamber and some in the white chamber. Each conditioning chamber was 14 cm × 14 cm × 22 cm and differed in visual and tactile cues. One chamber had black walls with a stainless-steel grid floor, while the opposite chamber had white walls with a wire mesh floor. A neutral central chamber separated the conditioning chambers and allowed access via guillotine doors. During the habitation and conditioning phases, mice had access to one of the solutions via a bottle protruding one inch inside the chamber from the right or left wall of one of the conditioning chambers (Supplementary Figure 1 and Supplementary Data). Mice were licking and drinking the solution, and behavior was recorded using overhead cameras, and time spent in each chamber was quantified using TopScan automated tracking software (Clever Systems, Inc.). The apparatus was cleaned with 70% ethanol followed by water between sessions. The paradigm consisted of 4 phases: Habituation, Conditioning, Extinction, and Reinstatement.

##### 2.3.1.1 Habituation phase

Male (*n* = 10) and female (11 mice) C57BL/6J mice were housed individually for a week to habituate to the novel environment. This experiment was repeated three times with 3–4 mice per cohort of male and female C57BL/6J mice. Mice were then conditioned with sugar solution in both chambers to habituate mice to the place conditioning apparatus and promote self-administration of sucrose (4%). On each conditioning day, mice were transported to the testing room and allowed to habituate to the room environment for 1 h before each session. Mice were then placed individually into one of the conditioning chambers with access to a 4% sucrose solution and allowed to adapt to the chamber and self-administer the solution for 60 min. The following day, mice were placed in the opposite chamber and allowed self-administer the sucrose solution for 60 min. The volume of each solution was measured at the start and end of each day during different phases of the CPP, except on the test days, when animals were tested under a drug-free state (no bottle present). The difference in the weight of the bottle was considered the volume consumed. We measured the volume of each solution consumed by each mouse daily. We also recorded their body weight. These two parameters were used to prepare the oxycodone solution. For example, if a mouse weighs 20 g and the volume consumed was 1 ml. The solution was made such that if a mouse consumes 1 ml of the oxycodone solution, it will yield a 16 mg/kg dose of the drug. First, we calculated the mg of oxycodone for a 20 g mouse (16 mg for 1000 g (16 mg/kg), how many mg for a 20 g mouse = 16 * 20/1000 = 0.32 mg). Thus, for this mouse, we prepared a 0.32 mg/ml solution and if the mouse drinks 1 ml of the solution, the dose will be 16 mg/kg. Mice received four conditioning each week for 2 weeks and then were tested for baseline place preference in a single 15 min session. On the baseline test day, mice were placed in the gray middle chamber with free access to all three chambers; the amount of time that mice spent in each chamber was recorded and analyzed to determine their baseline place preference and for the assignment of the sugar-paired chamber and oxycodone-paired chamber.

##### 2.3.1.2 Conditioning phase

During this phase, mice received conditioning while having access to oral oxycodone in 4% sucrose solution in one chamber and sucrose alone in the opposite chamber on alternating days. Conditioning sessions were conducted 4 days per week (two oxycodone-paired and two sucrose-paired sessions per week) for 2 weeks. During each 60-min session, mice were confined to the assigned chamber for 60 min. A conditioned place preference (CPP) test was conducted 24 h after the final conditioning session of each week under a drug-free state. We tested mice for CPP under a drug-free state because we were concerned that the sugar solution intake would be preferred if a choice was given between the two solutions, as seen during the conditioning particularly in mice lacking mu as well as female mice, although mice had access to only one of the solutions on each day. On the place preference test day, mice were placed in the central neutral chamber and allowed to explore the conditioning chambers for 15 min, and the amount of time that mice spent in each chamber was recorded.

##### 2.3.1.3 Extinction phase

To assess extinction of oral oxycodone-induced conditioned place preference, mice were confined to the conditioning chambers while having access to only the sucrose solution on each extinction training day. Mice received extinction training 4 days each week for a total of 2 weeks unless indicated. Mice were tested for the extinction of CPP 24 h following the last extinction training of each extinction training set. Extinction was defined as a significant reduction in the amount of time that mice spent in the oxycodone-paired chamber on the previous week(s) or no difference between the amount of time that mice spent in the oxycodone-paired vs. sugar-paired chamber.

##### 2.3.1.4 Reinstatement phase

To assess reinstatement of oral oxycodone-induced conditioned place preference, mice were transported to the testing room and allowed to habituate for 1 h in the test room. Mice were then injected with oxycodone (5 mg/kg, i.p.), placed in the central neutral chamber, and the amount of time that mice spent in each chamber was recorded for 15 min to assess the reinstatement of CPP.

#### 2.3.2 The role of MOP in oxycodone-induced CPP, its extinction and reinstatement

Activation of MOPs mediates the primary rewarding effects of opioids. The MOPs are found in the brain areas involved in reward, motivation, and learning, such as the ventral tegmental area, nucleus accumbens, hippocampus, and related limbic structures [18; 19; 20]. It is proposed that activation of MOPs inhibits γ-aminobutyric acid-ergic (GABAergic) interneurons in mesolimbic circuits, thereby reducing inhibition of dopaminergic neurons by GABA and increasing the amount of dopamine released to downstream targets. The process of increased dopamine release facilitates reinforcement learning and the formation of drug-context associations that can lead to sustained drug-seeking behavior (Fields and Margolis, 2015). Genetic deletion of the MOP eliminated many of the rewarding and reinforcing effects of opioids, demonstrating that the activation of the MOP is essential for opioid-induced behaviors (Matthes et al., 1996). Therefore, we used mice lacking MOP and their wild-type littermates to further validate our mouse model of oxycodone self-administration and reward. The procedure was the same as described above, except in this experiment we used male mice lacking MOP and their wild-type littermates (*n* = 5 mice per genotype) and compared sugar and oxycodone intakes and the CPP response in mice of both genotypes. This experiment was repeated two times with 2–3 mice per genotype in each cohort.

### 2.4 Statistical analysis

Data represent the mean ± SEM of the volume of solution intake and the amount of time that mice spent in the conditioning chambers on each test day and were analyzed using repeated-measures analysis of variance (ANOVA) with the conditioning days or test days as the within-subjects factor and the time in the oxycodone-paired vs. vehicle-paired chambers on each test day or the volume intake in each chamber, genotype as between-subjects factors where appropriate. To test for vigor and reproducibility, each experiment was conducted at least twice with 4 mice per cohort, except the experiment with MOP mice (2–3 mice/genotype per cohort). Significant interactions were followed by *post hoc* comparisons, and statistical significance was set at *P* < 0.05.

## 3 Results

### 3.1 Male C57BL/6J mice orally self-administered oxycodone in a sucrose solution and acquired a significant conditioned place preference (CPP) response

**FIGURE 1:**
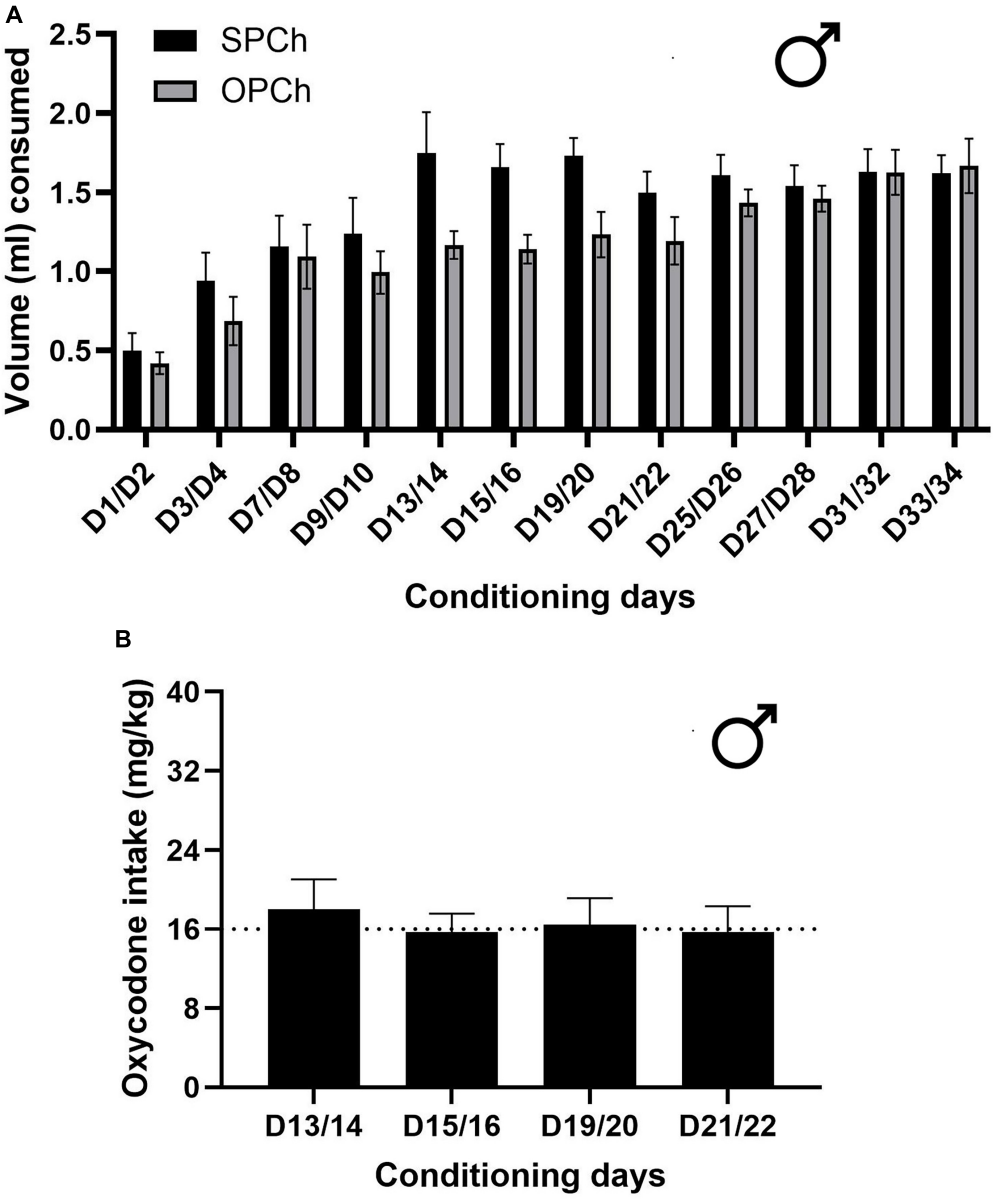
Volume of oxycodone and sugar intake (ml) during the habitation, conditioning and extinction phases **(A)** and oxycodone consumption (mg/kg) during the conditioning phase **(B)** in male C57BL/6J mice (*n* = 10). Results are presented as the mean (± SEM) of volume of sucrose (4%) and/or oxycodone (in 4% sucrose) solution consumed during the habituation, conditioning and extinction training sessions **(A)** and intake of oxycodone (mg/kg) on the conditioning days **(B).**

We combined oral oxycodone self-administration with the place conditioning paradigm to evaluate whether orally self-administered oxycodone would induce a conditioned place preference (CPP) in male C57BL/6J mice. The protocol consisted of a habituation phase during which animals were conditioned with sugar (4% sucrose) in both chambers. This phase habituated the mice to the apparatus and the conditioning chambers, as well as to promote sugar intake. This phase lasted for 2 weeks and two sessions in each chamber per week. Mice were then tested for a baseline (BL) place preference, also known as the preconditioning phase. Mice were then received conditioning with sucrose (4%) in one chamber (called the sucrose-paired chamber), and access to oxycodone in 4% sucrose solution in the opposite chamber (called the oxycodone-paired chamber). This phase also lasted for 2 weeks and two conditioning with sugar in one chamber and two conditioning with oxycodone in the opposite chamber per week. Mice were then tested for place preference 24 h after the last conditioning each week (Test1 for week 1 and Test2 for week 2). Mice were then exposed to extinction training for the next 2 weeks, having access to sugar only in each conditioning chamber. On day 5 of each week, mice were tested for extinction (Ext1 on the first week and Ext2 on the second week). Mice were then tested for reinstatement of CPP, in which mice received a challenge dose of oxycodone (5 mg/kg, i.p., Oxy5). Figure 1A shows oral intake of oxycodone and sucrose on the conditioning days. A two-way repeated measures ANOVA, where the factors are solution intake in the two contexts and day with conditioning days as the within factor, indicated a significant main effect of time (F_(5.10, 117.40)_ = 17.47, *P* < 0.0001), indicating that intake was increased significantly across conditioning days compared to the first conditioning day in each chamber. However, there was no significant main effect of context (*F*_(1, 23)_ = 3.07, *P* > 0.05) or interaction between context and time, suggesting that no overall difference existed in sugar vs. oxycodone consumption except on days 15/16 and 19/20 (Figure 1A). Furthermore, on average, mice consumed the designated dose of oxycodone (Figure 1B). As can be observed, on average, male C57BL/6J mice consumed about 16 mg/kg (shown by a broken line) each day.

**FIGURE 2:**
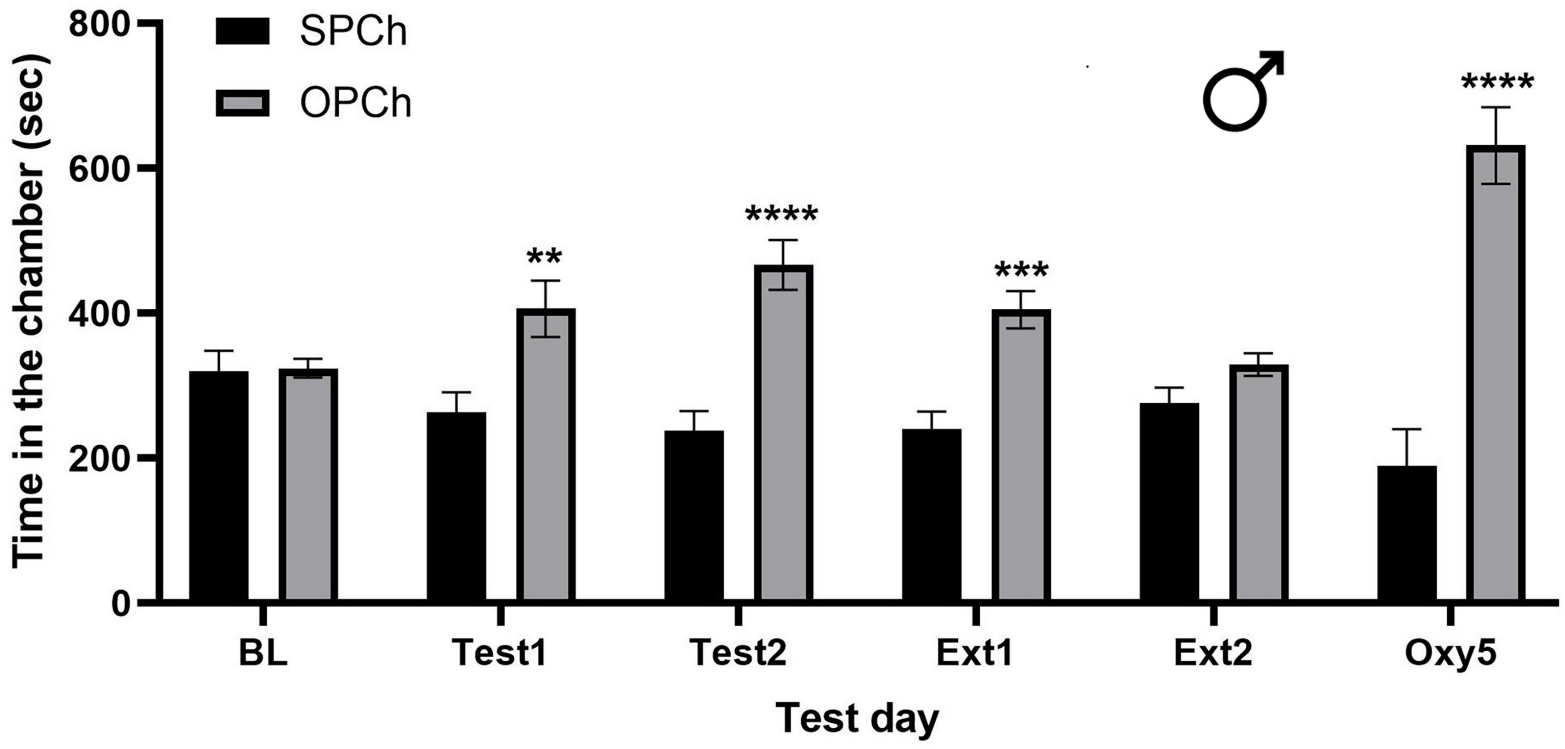
CPP following oxycodone self-administration in male C57BL/6J mice. Results represent the mean ( ± SEM) of the amount of time that mice spent in the sucrose-paired chamber (SPCh) or oxycodone-paired chamber (OPCh) on each test day. BL, baseline; Test1 and Test2, place preference tests following the first and second set of oxycodone conditioning, respectively; EXT1, EXT2, test for extinction following the first and second extinction training; Oxy5, Reinstatement following a challenge dose of oxycodone (5 mg/kg, i.p.); **P < 0.01, ***P < 0.001, ****P < 0.0001 vs. SPCh.

Oral oxycodone self-administration induces conditioned place preference (CPP) in male C57BL/6J mice (Figure 2). Two-way repeated measure ANOVA, where context and phases of the place conditioning paradigm as the between factors and test days as the within factor, revealed a significant effect of time (*F*_(2.35_, _42.24)_ = 3.40; *P* < 0.05), context (F_(1, 18)_ = 44.81, *P* < 0.0001) and a significant time x context interaction (*F*_(2.35, 42.24)_ = 14.17; *P <* 0.0001), indicating that mice changed their preference toward the chamber paired with oxycodone during the conditioning days. There was no initial preference toward one over the other conditioning chamber, confirming that an unbiased paradigm was used in the present study. After the first conditioning session (Test1), mice spent more time in the oxycodone-paired chamber over sucrose-paired chamber (*P* < 0.01). Following the second set of conditioning and the test for place preference (Test2), mice showed a robust and significant CPP, i.e., spending significantly more time in the oxycodone-paired chamber than the sucrose-paired chamber (*P* < 0.0001), confirming acquisition of an oxycodone-context association. Mice still exhibited a greater preference toward the oxycodone-paired chamber over sucrose-paired chamber following the first extinction test (Ext1). Nevertheless, following the second extinction training, mice displayed extinction, as there was no significant difference in the amount of time that mice spent in the oxycodone-paired chamber vs. sucrose-paired chamber (Figure 2, Ext2, *P* > 0.05). Upon re-exposure to oxycodone during the reinstatement test (Oxy5), mice showed a significant preference for the oxycodone-paired chamber over the sucrose-paired chamber and for the oxycodone-paired chamber on the reinstatement day than Ex2 (*P* < 0.0001). These results indicate that oral oxycodone conditioning in male C57BL/6J mice induces a significant CPP that is sensitive to extinction and can be reinstated following oxycodone re-exposure.

### 3.2 Mice lacking MOP failed to exhibit CPP

**FIGURE 3:**
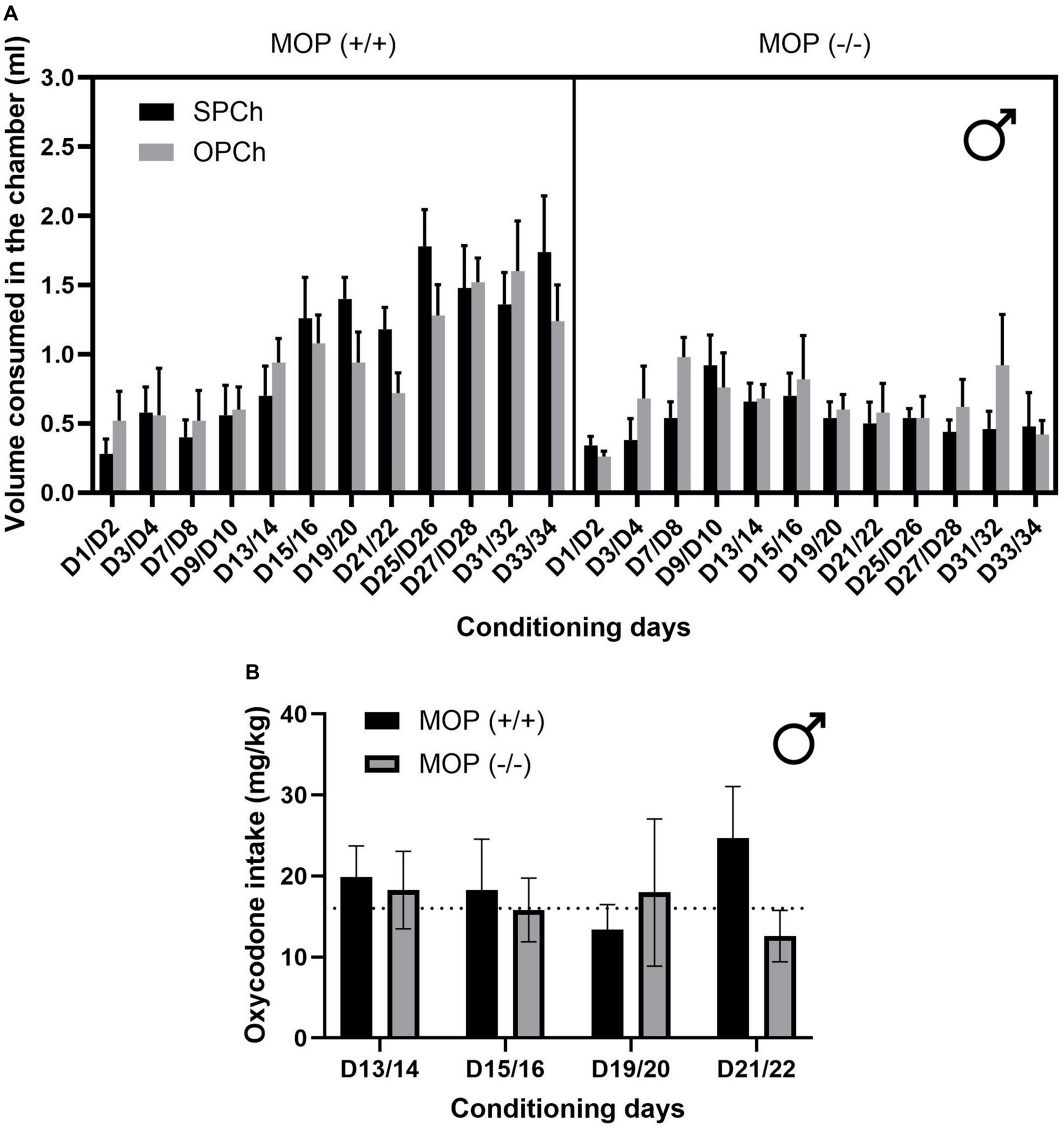
Volume of oxycodone and sugar self-administered during the habituation, conditioning and extinction training sessions **(A)** and oxycodone intake (mg/kg) during the oxycodone conditioning days (3B) in male mice lacking MOP (MOP-/-) and their wild-type (MOP+/+) controls (*n* = 5 mice per genotype). Results are the mean ( ± SEM) of sucrose (4%) and oxycodone (in 4% sucrose) intake during the habituation, conditioning and extinction sessions as well the dose of oxycodone (mg/kg) that mice of the two genotypes consumed **(B)** on the oxycodone conditioning days.

We quantified intake patterns of sucrose and oxycodone in male mice lacking MOP and their wild-type littermates during the conditioning phase as well as during the extinction training sessions (Figure 3A). Three-way repeated measure ANOVA, where genotype, solution type and day were the between factors, and conditioning days as the within factor, revealed a significant effect of time (*F*_(11,96)_ = 3.43; *P* < 0.001), genotype (*F*_(1,96)_ = 34.34; *P* < 0.0001) as well as day x genotype (*F*_(1,96)_ = 3.81; *P* < 0.0001) and genotype x context (*F*_(1,96)_ = 4.76; *P* < 0.05) interactions with no other factors and interactions being significant (*P* > 0.05). *Post hoc* comparisons did not show any significant difference between intake in the sucrose-paired chamber and oxycodone-paired chamber for mice of either genotype during the habituation phase (*P* > 0.05). Although intake was reduced in mice lacking MOP, there was no difference in solution intake between the two chambers on the oxycodone conditioning days. Also, the dose of oxycodone calculated based on this reduced intake was comparable to that in wild-type mice (Figure 3B). However, there was an increase in sugar intake in wild-type mice compared to knockout mice (*P* < 0.05). Overall, these results show that wild-type mice consumed sugar and oxycodone comparably, but the intake of oxycodone or sugar solution was higher at times in wild-type mice than in mice lacking MOP. Furthermore, sugar intake was lowered in mice lacking MOP, showing that the sugar reinforcement is reduced in the absence of MOP signaling.

**FIGURE 4:**
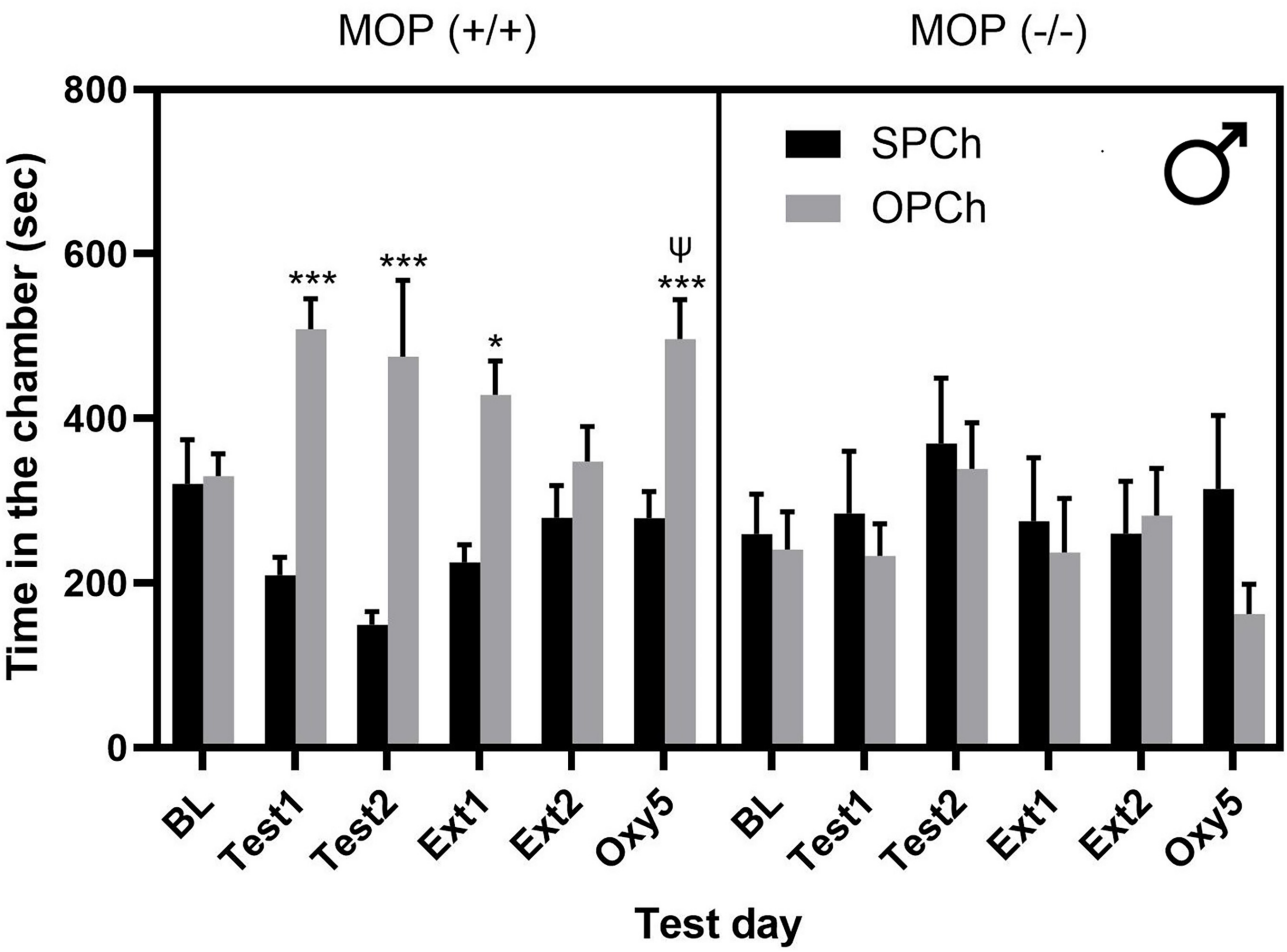
CPP following oxycodone self-administration in male mice lacking MOP and their wild-type littermates. Results represent the mean ( ± SEM) of the amount of time that mice spent in the sucrose-paired chamber (SPCh) or oxycodone-paired chamber (OPCh) on each test day. BL, baseline; Test1 and Test2, place preference tests following the first and second set of oxycodone conditioning, respectively; Ext1, Ext2, test for extinction following the first and second extinction training; Oxy5, Reinstatement following a challenge dose of oxycodone (5 mg/kg, i.p.); *P < 0.05, ***P < 0.001 vs. SPCh in MOP+/+ mice; ^Ψ^ *P* < 0.05 vs. OPCh of MOP-/-.

Figure 4 shows oral oxycodone self-administration induced CPP, and this response was dependent on MOPs. A three-way repeated measures ANOVA, with genotype, context and phases of the place conditioning paradigm were as the between factors and test day as the within factor, revealed no significant main effect of time (*F*_(5,48)_ = 0.51; *P* > 0.05), but there were significant main effects of genotype (*F*_(1,48)_ = 11.09, *P* < 0.003) and context (*F*_(1,48)_ = 8.516; *P* < 0.01), as well as a significant genotype x context interaction (*F*_(1,48)_ = 22.63, *p* < 0.0001), suggesting that oxycodone induced CPP and this response was genotype-dependent. No other interaction terms demonstrated statistical significance in our study (*P* > 0.05). *Post hoc* analysis showed that mice of each genotype showed no preference for the drug- vs. vehicle-paired chambers during the initial preference test (BL), indicating that we used an unbiased place conditioning paradigm. After oxycodone conditioning, wild-type mice demonstrated a robust CPP such that they spent significantly more time in the oxycodone-paired chamber when compared to the vehicle-paired chamber in Test1 (*P* < 0.05) and again in Test2 (*P* < 0.01). In contrast, as expected, MOP knockout mice did not develop CPP, as the amount of time that mice spent in the two chambers was statistically indistinguishable from each other (*P* > 0.05). During Ext1 and Ext2, wild-type animals gradually lost their CPP. Although they still showed a significant difference in preference on the first extinction test day (Ext1; *P* < 0.05), this difference in preference was eliminated by Ext2, indicating successful extinction in wild-type mice. The oxycodone challenge reinstated CPP in wild-type mice (*P* < 0.001), while the knockout mice did not exhibit reinstatement and instead showed aversion, spending significantly less time in the oxycodone-paired chamber when compared to the vehicle-paired side and compared to the oxycodone-paired chamber in wild-type controls (*p* < 0.001). Our results indicate that oral oxycodone self-administration induced reward-associated memory that is acquired, extinguished, and reinstated in a MOP-dependent manner.

### 3.3 Female mice also expressed CPP, which was extinguished and reinstated

**FIGURE 5:**
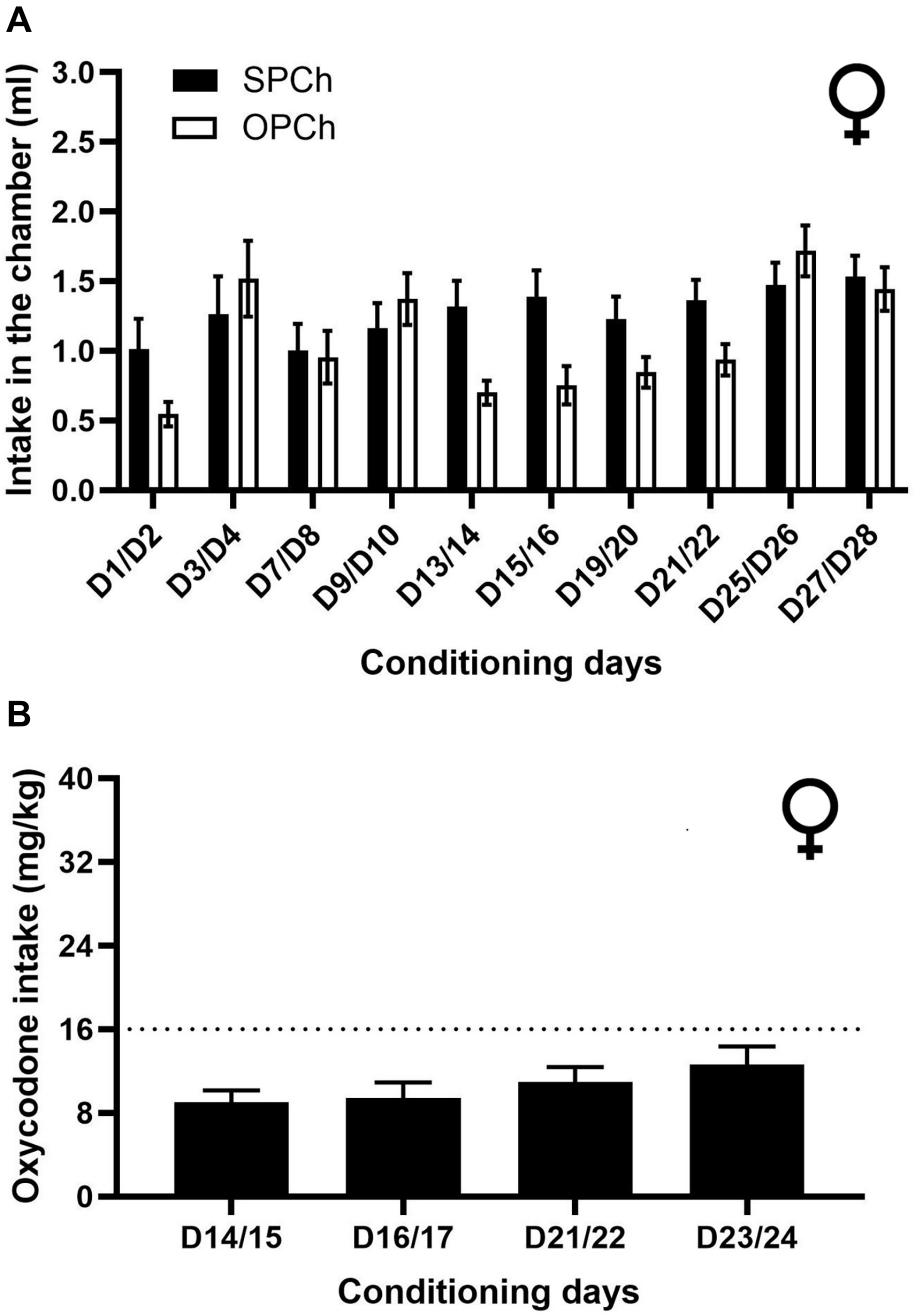
Oxycodone and sugar self-administration (ml) during the habituation, conditioning, and extinction days **(A)** and oxycodone intake during the conditioning days **(B)** in female C57BL/6J mice (*n* = 11). Results are representative of the mean (± SEM) of sucrose (4%) and oxycodone (in 4% sucrose) intake during habituation, conditioning and extinction training sessions **(A)** and oxycodone dose **(B)**.

Figure 5A shows the consumption of sugar and oxycodone in female mice in each conditioning chamber during the entire time course of the study. A two-way repeated measures ANOVA, with solution intake in the two contexts and day as the between factors and conditioning days as the within factor, revealed a significant main effect of time (*F*_(3.02, 60.41)_ = 6.84, *P* < 0.001), and time x solution interaction (*F*_3.02, 60.41)_ = 3.28, *P* < 0.05), suggesting that mice were able to vary their intake across the conditioning days. However, there was no significant main effect of solution (*F*_(1, 20)_ = 1.43, *P* > 0.05), showing that mice generally did not prefer one solution over the other during habituation and extinction sessions. The *Post hoc* test, however, revealed a significant reduction in oxycodone compared to sugar intake on the conditioning days 13/14 – 19/20 (*P* < 0.05). Oxycodone dose calculation also revealed that female mice consumed a lower dose of oxycodone on the first 2 days but slowly increased their intake leading to a dose close to the appropriate dose on the last conditioning day (Figure 5B; the expected dose is shown by a broken line).

**FIGURE 6:**
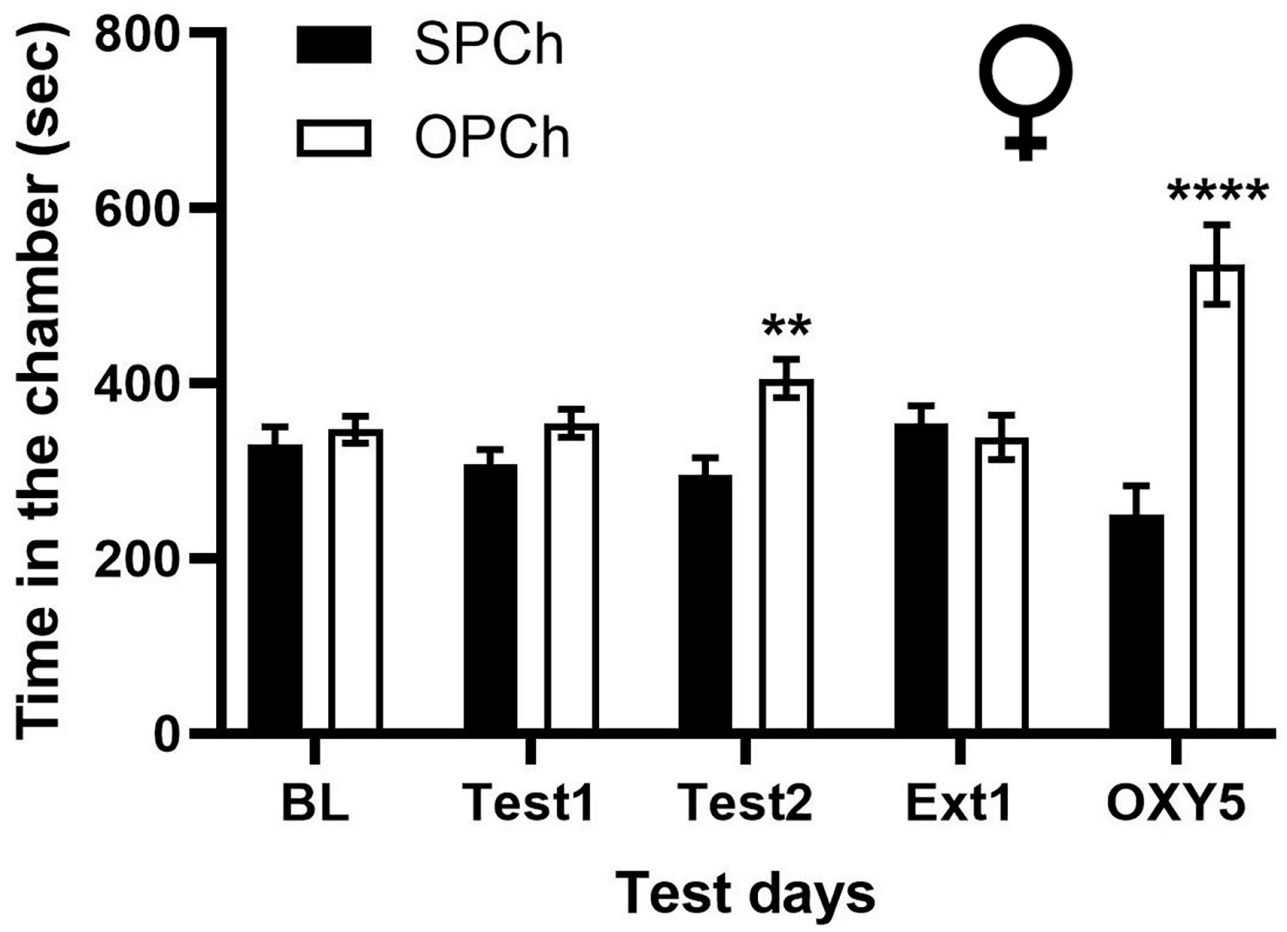
CPP following oxycodone self-administration in female C57BL/6J mice. Results demonstrate the mean ( ± SEM) of the amount of time that mice spent in the sucrose-paired chamber (SPCh) or oxycodone-paired chamber (OPCh) on each test day. BL, baseline; Test1 and Test2, place preference tests following the first and second set of oxycodone conditioning, respectively; Ext1, test for extinction following the first extinction training; Oxy5, Reinstatement following a challenge dose of oxycodone (5 mg/kg, i.p.); **P < 0.01; ****P < 0.0001 vs. SPCh.

Oral oxycodone self-administration induced a significant CPP in female mice but only following the second set of conditioning with oxycodone, a response was more prone to extinction, as it extinguished upon the first extinction training, but the reinstatement was robust (Figure 6). Two-way repeated measures ANOVA, with context and different phases of the place conditioning paradigm as the between factors and test days as the within factor, indicated that there was no main effect of time (*F*_(1.91, 38.14)_ = 2.90, *p* = 0.07), however, there was a significant main effect of context (*F*_(1,20)_ = 13.21, *P* < 0.01), and a significant phases of conditioning x context interaction (*F*_(1.91, 38.14)_ = 17.32, *P* < 0.0001). *Post hoc* analysis showed that female mice spent a comparable amount of time in the two chambers on the baseline test day (BL) and following the first set of oxycodone conditioning (Test1), but preference was shifted for the oxycodone-paired side following the second set of oxycodone conditioning (Test2; *P* < 0.05), indicating that females formed a drug-context association following the second set of oxycodone self-administration and conditioning. Also, female mice exhibited extinction on the first set of extinction training (*P* > 0.05). However, female mice exhibited a robust CPP after re-exposure to oxycodone (*P* < 0.001). These results indicate that oral oxycodone self-administration induces CPP in female mice that can be extinguished and reinstated.

## 4 Discussion

The main findings of the current study are that oral self-administration of oxycodone by male and female C57BL/6J mice was found to induce a conditioned place preference (CPP) response that was extinguished upon removing oxycodone from the solution during the extinction training and reinstated after re-exposure to oxycodone. The CPP response was completely abolished in mice lacking MOP, suggesting that the rewarding action of oxycodone is via MOP. Together, our results indicate that our mouse model of reward using oral self-administration of oxycodone in a sweetened solution (4% sucrose) is a valid and translationally relevant method for studying the underlying mechanisms of oxycodone’s reward and reinforcement in the same animal.

Oxycodone is an opioid agonist used to treat pain in patients, but it is also abused due to its rewarding and reinforcing action (Babb et al., 2023; Brice-Tutt et al., 2023; Collins et al., 2016; Fan et al., 2019; Niikura et al., 2013; Socha et al., 2024), as well as its discriminative stimulus in C57BL/6J mice (Walentiny et al., 2019) and non-human primates (Withey et al., 2020). In recreational opioid users, intravenous oxycodone has been associated with liking and high (Backonja et al., 2016), indicating that oxycodone possesses abuse potential. Considering that the main route of administration of the drug is oral, we assessed whether voluntary oral oxycodone self-administration would induce reward in mice. Our results showed that male mice self-administered oxycodone and exhibited a robust CPP even after two one-hour oxycodone conditioning. This response was extinguished when oxycodone was replaced with sugar, showing that sugar alone does not induce CPP, and this response is mainly due to oxycodone conditioning. However, we cannot rule out the possibility that the CPP response could have been due to a combination of oxycodone and sucrose. Thus, further research is needed to delineate between the two.

Next, we examined whether this response is mediated via MOP, as oxycodone is considered a MOP agonist with some activity at the kappa opioid receptors (KOP). Therefore, we used mice lacking MOP and their wild-type littermates to determine the role of MOP in the CPP response induced by oral oxycodone self-administration. Previous studies have demonstrated that MOP knockout mice do not demonstrate CPP and exhibit aversion-like responses when challenged with morphine or morphine-6-glucuronide (Nguyen A. T. et al., 2012). Our results confirmed that MOP signaling is necessary for oxycodone reward, utilizing a novel mouse model of voluntary oral oxycodone self-administration in a sweetened vehicle and extending prior studies using oral drug self-administration in mice and rats (Enga et al., 2016; Jimenez et al., 2017). Interestingly, MOP knockout mice expressed aversion toward the oxycodone-paired chamber on the reinstatement test day, as we observed with morphine or morphine-6-glucuronide (Nguyen A. T. et al., 2012). The aversion-like response seen in MOP knockout mice may result from activation of non-MOP receptors. Oxycodone can activate KOP, which has been linked to aversion in rodents and dysphoria in humans (Nguyen A. T. et al., 2012). Thus, future studies are needed to determine the underlying mechanisms of such an aversive response that develops in the absence of MOP following injection of oxycodone and morphine. Together, these results indicate that our mouse model of reward following oral self-administration of oxycodone in a sweetened solution is a valid and translationally relevant method to use in preclinical studies to characterize the underlying mechanisms of oxycodone’s reward and reinforcement in the same animal. It is worth noting that mice lacking MOP consumed less sucrose than wild-type mice, but we prepared the dose of oxycodone based on the volume consumed and the weight of each animal. We did not find any significant difference in oxycodone dosing between wild-type and knockout mice (Figure 3B). Thus, the lack of CPP in knockout mice is probably not due to differences in oxycodone dose.

Sex differences exist and are another biological variable that influences opioid sensitivity and reward-related behavior. Both clinical and preclinical data demonstrate that males and females exhibit differences in the rewarding effects of opioids, the development of opioid dependence, and the susceptibility to relapse after discontinuing opioid use (Babb et al., 2023; Brynildsen et al., 2020; Knauss et al., 2023). Differences in the action of opioids between males and females may be attributed to the fact that the activity of the MOP is modulated by sex hormones. For example, estrogen increases Oprm1 gene expression and thus the number of available MOPs in reward-relevant brain areas (Becker and Koob, 2016), while testosterone decreases the number of available MOPs and enhances MOP desensitization (Becker and Koob, 2016). Despite the male/female differences in opioid reward, only a small number of studies have evaluated sex differences in CPP after voluntary oral ingestion of oxycodone. Prior evidence suggests not only sex-related differences in reward but also relapse susceptibility and highlights the need to include sex as a biological variable in all addiction research (Becker and Koob, 2016). Thus, we also examined whether female mice would self-administer oxycodone and if this would result in a CPP response. Our results revealed that female mice exhibited a CPP response that was extinguished and reinstated. Although at first glance there appeared to be differences in the emergence of the CPP response and its extinction between male and female mice, we did not compare and statistically analyze these changes between male and female mice, as oxycodone intake was reduced in female mice compared to male mice, leading to dosing inequality between male and female mice. Indeed, female mice consumed a reduced volume of the oxycodone solution but appeared to increase their intake from about 50% to 75% of the dose on the last conditioning (Figure 5B). This may be the reason that females did not exhibit CPP after the first set of conditioning with oxycodone, and the CPP response decayed faster. Thus, future dose-response studies should be conducted to assess sex-related differences in the acquisition, extinction, and reinstatement of the CPP response following oral self-administration of oxycodone.

In summary, this research has shown that mice can develop a strong CPP following voluntary oral oxycodone self-administration. The development of CPP was dependent upon exposure to the drug (acquisition), eliminated with repeated non-drug exposures (extinction), and reinstated when mice were exposed to the drug again. The effects were completely dependent on MOP because no CPP could be observed, nor could reinstatement occur in mice lacking MOPs. These results provide evidence that the current paradigm is a valid model for studying the rewarding effects of oxycodone and relapse-related behavior that can be translated into the clinic to help understand how to prevent and treat opioid addiction. Oral administration represents the most common first method by which people misuse prescription opioids. Thus, the current mouse model is the first study providing evidence for the development of a translational mouse model of reward utilizing oral oxycodone self-administration. This model can be used for investigating the mechanisms underlying oxycodone reward and reinforcement following its oral self-administration. This model can also be used to characterize the underlying mechanisms of extinction and reinstatement. In the future, the application of these paradigms will allow researchers to identify and validate receptor-specific targets for the treatment of opioid use disorders.

## Data Availability

The original contributions presented in the study are included in the article/Supplementary material, further inquiries can be directed to the corresponding author/s.
