# Supplementary material for: A mouse model of oxycodone reward following its oral self-administration in a sweetened solution
Source: Front Behav Neurosci. 2026 Jul 27;20:1862996. doi: 10.3389/fnbeh.2026.1862996 (PMC13454296; doi:10.3389/fnbeh.2026.1862996)

Supplemental Data

We used the place conditioning paradigm to determine whether voluntary oral oxycodone intake would produce conditioned place preference (CPP), a model of reward [1; 2; 3]. Figure 1S illustrates the place conditioning apparatus that we used in this study. As shown, the apparatus is made of three chambers: a central and two side chambers, which are used for conditioning the animals. The conditioning chambers are decorated black and white with mesh floor. On the conditioning days, a bottle containing one of the solutions (4% sucrose or oxycodone in 4% sucrose solution) is inserted through a hole created on the right or left wall of one of the conditioning chambers and the sipper tube protruding one inch inside each chamber. Animals had free access to one of the solutions via licking the sipper tubes. The weight of the bottle was calculated at the start and end of conditioning on each day. The difference between the two measurements was used a the volume consumed by each mouse.

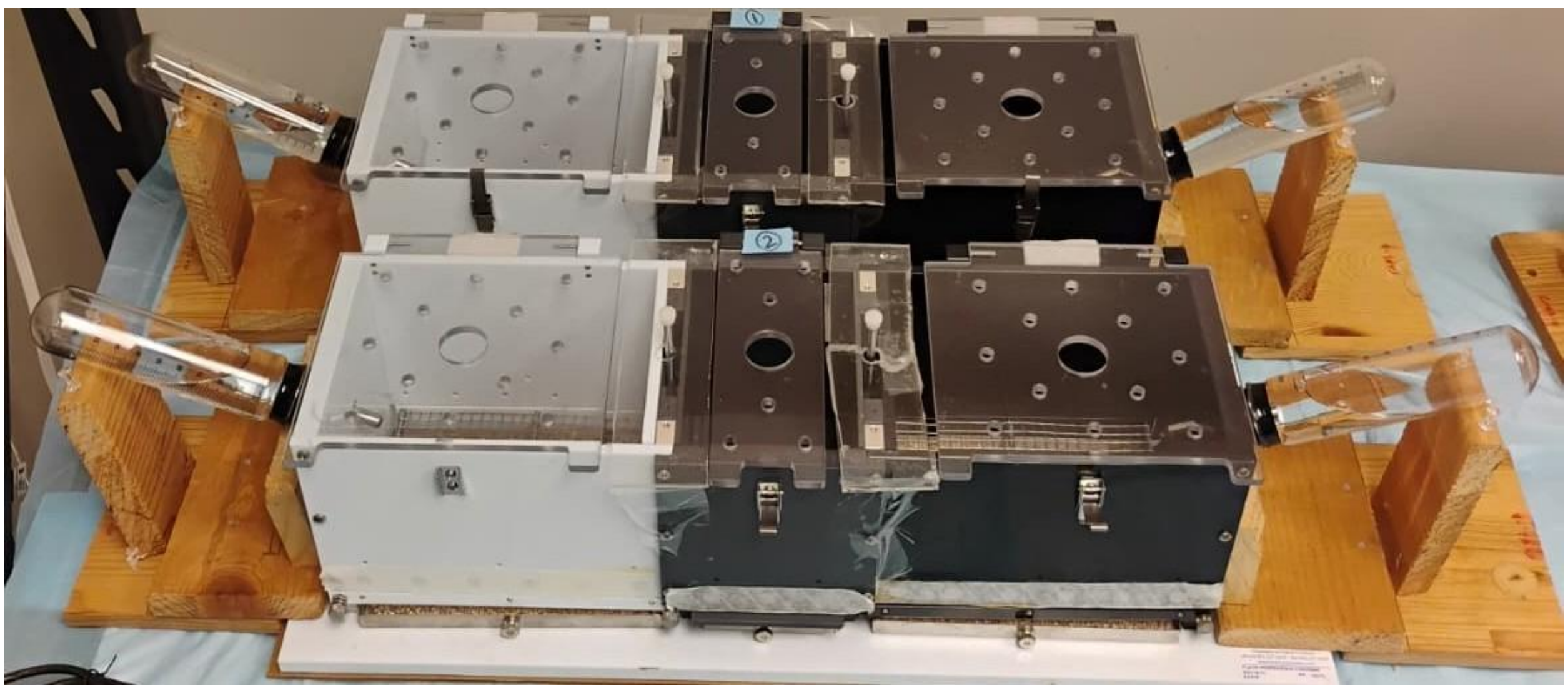

Supplement: Supplementary file 1 [file Data_Sheet_1.pdf]
